# Reduction of Free Fatty Acids in Waste Frying Oil Using Thermally Treated Mortar

**DOI:** 10.1021/acsomega.6c03842

**Published:** 2026-07-08

**Authors:** Laís Fernanda Juchem do Nascimento, Joel Gustavo Teleken, Fabiano Bisinella Scheufele, Thompson Ricardo Weiser Meier, Paulo André Cremonez, Rodrigo Brackmann

**Affiliations:** † 646047 Federal University of Paraná, Department of Engineering and Exact Sciences, Palotina 80060-000, Brazil; ‡ Federal University of Technology−Paraná, Department of Bioprocess and Biotechnology Engineering, Toledo 85902-490, Brazil; § Federal University of Technology−Paraná, Department of Chemical Processes, Toledo 85902-490, Brazil; ∥ Federal University of Technology−Paraná, Department of Chemistry, Pato Branco 85503-390, Brazil

## Abstract

Amid growing global efforts to mitigate climate change and decarbonize energy systems, biodiesel derived from waste cooking oils (WCOs) has emerged as a sustainable and economically viable alternative. However, the high free fatty acid (FFA) content in WCOs presents a significant barrier to efficient transesterification, requiring cost-intensive pretreatment stages. To address this challenge, thermally treated mortar waste from construction and demolition activities was evaluated as a low-cost adsorbent for reducing the acidity of WCOs collected from a university food service facility. Material characterization by scanning electron microscopy coupled with energy-dispersive X-ray spectroscopy (SEM-EDS) and Fourier transform infrared spectroscopy (FTIR) revealed that thermal treatment at 850 °C enhances surface porosity and increases the presence of basic calcium oxide (CaO) phases, facilitating FFA adsorption. The process achieved a 78% reduction in acid value, reaching approximately 0.55 mg KOH.g^–1^, which is below the typical threshold required for efficient transesterification. These results were obtained using a Central Composite Rotatable Design (CCRD) with a total of 17 experimental runs, including triplicates at the central point. The statistical model yielded a coefficient of determination (*R* ^2^) of 0.91. Under optimized conditions (20.29 °C, 153.53 rpm, 0.5 g of adsorbent), an acid value reduction of 78% and an adsorption capacity of 81.67 mg.g^–1^ were achieved. Kinetic modeling showed the best fit with the pseudo-second-order (PSO) model (*R* ^2^ = 0.97893), indicating a chemisorption-dominated process. Potential limitations include calcium leaching into the oil and the high energy demand associated with the calcination process.

## Graphical abstract

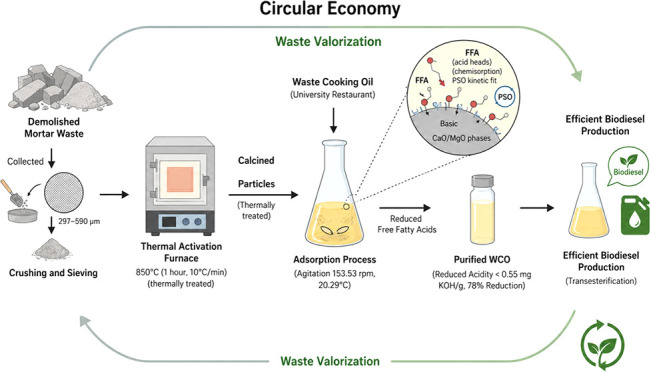

## 1 Introduction

Global CO_2_ emissions from the energy sector increased by 1.3% in 2023, reaching 40.6 GtCO_2_, the highest value in history, while atmospheric concentrations reached 419.31 ppm.^1^ Conjunctural factors, such as energy prices and geopolitical risk, affected emissions between 2021 and 2023 in distinct sectoral ways.^2^ In the United States, the second-largest emitter in 2022 (4.8 GtCO_2_), meeting the 50–52% reduction target by 2030 will require significant improvements in the energy transition and ecological innovation.^3^ In this context of environmental and energy crisis, the reuse of waste, specifically WCOs, emerges as a promising economic alternative and a viable raw material for biofuels.^4^ Improper disposal of WCO can harm the environment by clogging sewer systems and contaminating soil and water.^5–7^ However, WCOs with high FFA content cannot be directly converted into biodiesel through transesterification with methanol.^8^ To enable their use as feedstock, a preliminary esterification step is typically conducted using homogeneous acid catalysts, such as sulfuric acid, to reduce the FFA content. This reaction is usually followed by neutralization and catalyst separation processes.^9^ After pretreatment, the resulting oil generally exhibits an acid value below 2%,^10^ allowing the subsequent transesterification reaction to be carried out using a homogeneous base catalyst.^11^ Despite its effectiveness, this process requires extended reaction times, which can increase overall biodiesel production costs.

Among the primary raw materials used in biodiesel production, WCOs stand out due to their low cost and wide availability. These oils, of vegetable or animal origin, are usually discarded after food preparation in households, restaurants, fast-food chains, and the food industry.^12^ However, the quality of these residues varies significantly, potentially compromising the process efficiency and fuel quality. Factors such as oil type, reuse frequency, cooking temperature and method, dietary habits, and storage conditions directly impact the physicochemical properties of the feedstock.^6^

A major challenge in using WCO lies in its acidity. High FFA levels interfere with the transesterification reaction, reducing the yield and, in some cases, making a one-step process unfeasible. Estimates indicate that a 5% increase in acidity can reduce the final yield by up to 5%, while simultaneously raising operational costs. According to Singh et al.,^13^ using cooking oil with high FFA content requires additional pretreatment and higher methanol ratios to achieve full conversion, increasing both energy demand and operational costs. Consequently, the transesterification step becomes the primary driver of emissions and expenses due to the intense energy demand in production and refining advances.^14^ Therefore, pretreatment is considered to be essential.

Among the alternatives, adsorption has been recognized as a technologically and economically viable solution. This process can be carried out using various adsorbent materials, such as bentonite, activated carbon, silica, or even lignocellulosic residues, which typically offer good removal capacity and low environmental impact.^15^ Adsorption works by adhering a substance (the adsorbate), in gas or liquid form, to the surface of a porous solid (the adsorbent).^16^ While activated carbon is the most widely used material in purification steps due to its high efficiency, its high production cost limits its industrial-scale application. Consequently, researchers are increasingly seeking alternative adsorbents that are cheaper, more sustainable, and still demonstrate comparable performance.^17^

In 2020, global solid-waste generation was estimated to be approximately 2.24 billion tons, reflecting the growing pressure of population growth and urbanization on waste management systems. Construction and demolition waste (CDW) accounts for a significant fraction of this total, estimated at no less than 30%,^18^ a trend expected to intensify with urban expansion in various regions.^19,20^ The quantity and composition of CDW vary considerably across countries, but recent studies identify China, the United States, and the European Union as the largest generators.^21,54^ CDW recovery rates also differ substantially depending on local contexts, ranging from 7% to 90%.^22^ Although nearly 75% of this waste has potential for value-added reuse, it is estimated that about 35% is still disposed of in landfills, representing both an environmental burden and a missed opportunity for circular economy advancement.^23^

Materials such as quicklime, leftover cement, and mortar have already been tested as heterogeneous catalysts in biodiesel production using WCOs.^24,25^ However, most studies in the literature focus primarily on the transesterification step itself. Few studies have explored using these inorganic residues to reduce the FFA content prior to the reaction.

This study aims to address this gap by proposing the direct use of thermally treated mortar waste as an acidity-reducing agent, focusing on the technical and economic feasibility of pretreating low-quality WCOs for the production of biodiesel.

## 2 Materials and Methods

Figure 1 outlines the methodological steps employed to evaluate the use of construction waste as an adsorbent for reducing FFA levels in WCOs.

**Figure 1:**
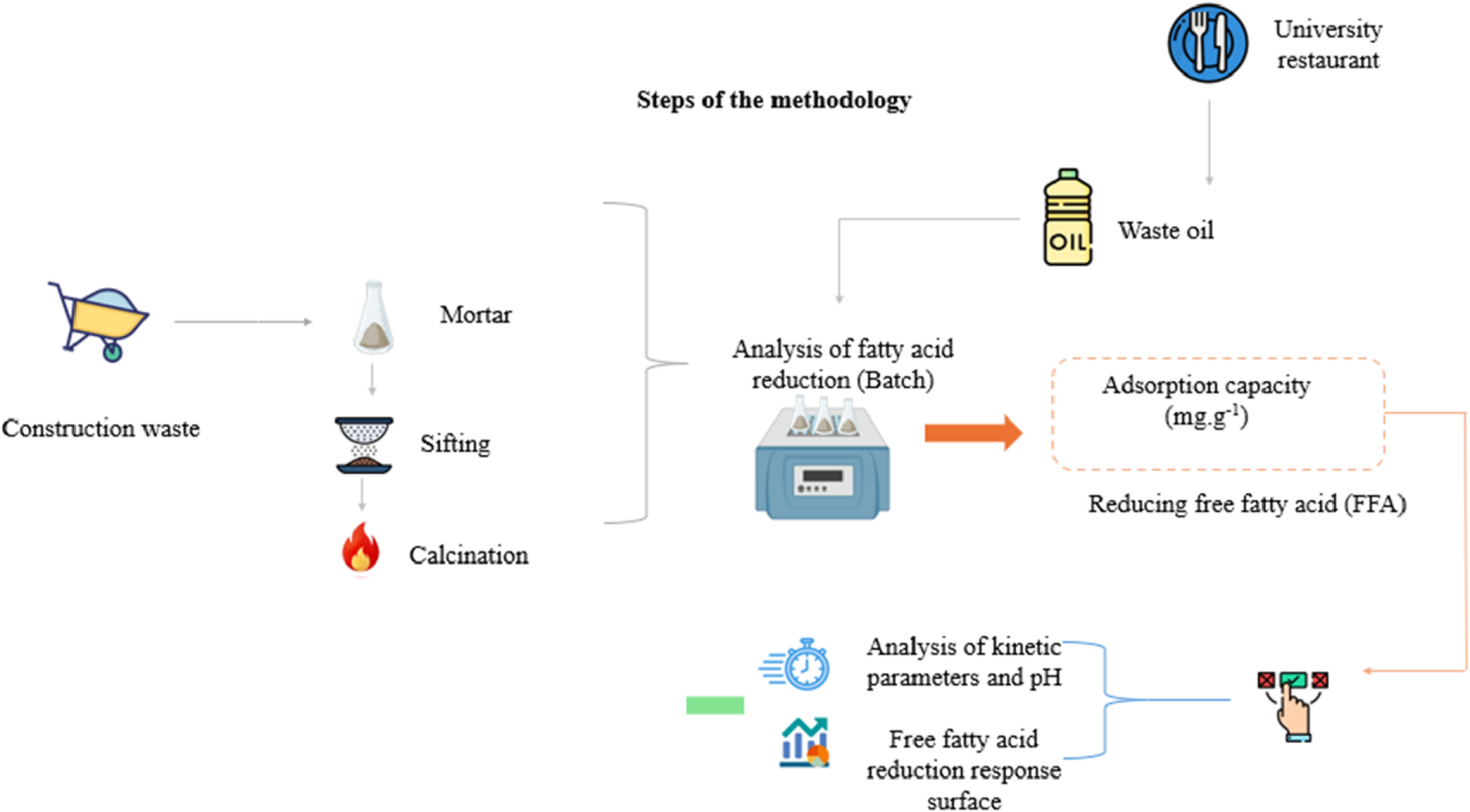
Methodological framework for adsorptive pretreatment using mortar waste created with BioRender.com.

### 2.1 Description of Materials and Reagents Utilized throughout the Study

The adsorbent used in this study was a mortar waste residue obtained from an actual construction site. The adsorbate used was WCO collected from the university restaurant at the Federal University of Paraná (UFPR).

The mortar was collected directly from the construction site, manually crushed, and sieved using a set of standard laboratory sieves to obtain particle size fraction between 590 and 297 μm. Subsequently, the material went through a thermal treatment in a muffle furnace at 850 °C, for 1 h, with a fixed heating rate of 10 °C min^–1^.^26^

### 2.2 Physicochemical Characterization

Thermogravimetric analysis (TGA) was conducted to determine the weight loss of the samples as a function of temperature. The analysis was perfomed under a nitrogen (N_2_) atmosphere from 25 to 900 °C at a heating rate of 10 °C min^–1^ under nonisothermal conditions. This allowed the identification of temperature ranges associated with the highest mass loss and the thermal decomposition behavior of the mortar samples.

To evaluate the structural and chemical changes induced by thermal treatment, we characterized the mortar residue before and after heating. The surface morphology was analyzed by scanning electron microscopy (SEM) using a TESCAN VEGA 3 microscope. To complement the microscopy analysis, energy-dispersive X-ray spectrometry (EDS) was performed using an energy-dispersive X-ray detector (PentaFET Precision) as a semiquantitative technique to determine the types and relative percentages of chemical elements present in the adsorbent samples.

Fourier transform infrared spectroscopy (FTIR) spectra were recorded from 4000 to 400 cm^–1^ using the KBr pellet method to identify functional groups. X-ray diffraction (XRD) analysis was performed using a Rigaku Miniflex 600 diffractometer equipped with Cu Kα radiation (λ = 0.154 nm). The equipment operated at 40 kV and 600 W. The diffraction patterns were recorded in the 2θ range of 5–85°, with a step size of 0.02° and a scanning rate of 0.8° min^–1^, allowing the identification of crystalline phases in the mortar samples.

### 2.3 Acidity Index Analysis

The adsorption experiments were conducted in batch mode by using a Dubnoff water bath for agitation. Equilibrium tests were performed in 250 mL Erlenmeyer flasks containing 33 g of WCO and 5 g of thermally treated mortar as the adsorbent. The WCO collected from the university restaurant presented an acid value of 2,539.31 mg KOH g^–1^. The adsorbent mass and agitation conditions were selected on the basis of the statistical design described in the next section. Experiments were performed for a fixed period of 2 h. The acid value was calculated using eqs 1 and 2, as presented in Table 1, which describe the relationship between the volume of titrant used and the free fatty acid content in the oil. After centrifugation, the samples were calculated for the acid value following the Adolfo Lutz Institute protocol (2008). For the best adsorption performance within 2 h, the adsorption capacity was calculated using eq 3. Adsorption kinetics were evaluated at time intervals of 5,15, 30, 90, 120, 180, 300, and 360 min.

**Table 1:** Equations Used for the Calculation of Acid Value

equations	parameters
1 $\mathrm{AV}=\frac{V\times C_{\mathrm{KOH}}\times M}{m}$	AV = acid value (mg KOH g^–1^ of oil); *V* = volume of KOH relative used in the titration (mL); *C* = concentration of the kOH solution (mol L^–1^); *M* = molar mass of NaOH (g mol^–1^); *m* = mass of oil (g)
2 $\%R=\frac{I_{Ai}-I_{Af}}{I_{Ai}}\times 100$	%*R* = percentage of acid value reduction
3 $q=\frac{V\left(C_{0}-C\right)}{m}$	where *q* is the amount of contaminant adsorbed per gram of adsorbent (mg g^–1^); *C*_0_ and *C* are the initial and final concentrations of the solution (mg L^–1^), respectively; *V* is the volume of the solution (L); and *m* is the mass of the adsorbent (g)

The acidity index was calculated in mg NaOH g^–1^ of the sample (eq 1). To convert this value to milligrams of potassium hydroxide (KOH), the result was multiplied by 1.4, since KOH has 1.4 times the molar mass of NaOH.

### 2.4 Experimental Design to Maximize the Reduction of the FFA Index

The analysis of kinetic curves allowed for the determination of the material performance in terms of FFA removal and the estimation of an appropriate contact time for the process. These results guided the configuration of subsequent experimental design. A central composite rotatable design (CCRD) was employed, utilizing statistical tools and a desirability function to help optimize the process conditions.

The CCRD considered three main variables: temperature, stirring speed, and mass of the mortar adsorbent. Each factor was tested at five levels (−1.68, −1, 0, 1, 1.68), as shown in Table 2. The experimental matrix consisted of a 2^3^ factorial design, supplemented by six axial points and three replicates at the central point, resulting in a total of 17 experimental runs. The central point was conducted in triplicate. The goal was to evaluate the efficiency of the system in reducing the FFA concentration in the WCOs and moisture.

**Table 2:** Levels of the Proposed Experimental Design (CCRD)

variables	–1.68	–1	0	1	1.68
bath temperature (°C)	15	17	20	23	25
bath agitation (rpm)	100	120	150	180	200
construction waste mass (g)	2	3.21	5	6.79	8

### 2.5 Analysis of Adsorption Mechanism: Kinetics and Isotherms

The treatment that showed the best average performance was selected for the kinetic pH analysis. The experimental data were fitted to nonlinear forms of the pseudo-first-order (PFO) and pseudo-second-order (PSO) models. The respective equations are presented in Table 3.

**Table 3:** Equations Used for Adsorption Capacity and Kinetic Modeling

equations	parameters
4 $\mathrm{PFO}:q_{t}=q_{e}\left(1-e^{-{k_{1}}^{t}}\right)$	*q*_e_ and *q_t_* (mg g^–1^) are the amounts adsorbed at equilibrium and at time *t*, respectively. *k*_1_ (min^–1^) is the rate constant of the pseudo-first-order model
5 $\mathrm{PSO}:q_{t}=\frac{k_{2}q_{et}^{2}}{\left(1+k_{2}q_{e}t\right)}$	*k*_2_ (g mg^–1^ min^–1^) is the rate constant of the pseudo-second-order model
6 $\mathrm{Elovich}:q_{t}=\frac{1}{\beta }\ln\left(\alpha \beta \right)+\frac{1}{\beta }\ln\left(t\right)$	α (mg g^–1^ min^–1^) is the initial adsorption rate, and β (g mg^–1^) is related to surface coverage and activation energy

To compare the application of different kinetic models, nonlinear regression was applied using the least-squares method. The quality of the fit was evaluated on the basis of the coefficient of determination (*R* ^2^) and the sum of squared errors (SSE). Initial parameters for each kinetic model were estimated from the experimental curves and refined by least-squares minimization, until convergence was achieved.

## 3 Results and Discussion

### 3.1 Chemical Composition of the Mortars

The EDS results, summarized in Table 4, corroborate the successful incorporation of calcium into the mortar matrix, resulting from the addition of quicklime during the synthesis stage. In its natural state, the material exhibits a composition predominantly composed of C–O–H species (62.49 wt %), along with significant fractions of Si (13.29 wt %) and Ca (12.85 wt %). This elemental distribution reflects a heterogeneous matrix composed of hydrated phases and silicate frameworks. Upon interaction with the oily medium, the mortar shows a substantial increase in C–O–H content (90.80 wt %), indicating the adsorption of organic compounds onto its surface. This change is accompanied by a marked decrease in Ca (3.70 wt %) and the complete depletion of Mg (0 wt %), suggesting that active basic sites were either masked by surface coverage or consumed through reactions with free fatty acids (FFAs). From a mechanistic perspective, the results indicate that thermal activation plays a key role in enhancing the material’s reactivity. After calcination, the significant increase in Ca (26.39 wt %) and Mg (7.71 wt %) suggests the formation of highly active CaO and MgO phases. These oxides provide strong basic sites capable of neutralizing FFAs through ionic interactions, as widely reported for calcined CaCO_3_-rich materials and MgO-based adsorbents.^27,28^

**Table 4:** Elemental Composition (wt %) of Mortar Samples in Natura, Thermally Treated (850 °C), and with Oil, Determined by EDS

element	Si	Ca	Ca/Si	Fe	C–O–H	Mg	Mg/Si	Al	K	Na	Ti	S	other elements
mortar in natura	13.29	12.85	0.96	1.65	62.49	3.82	0.29	4.11	1.15	0.27	0.37	0	0
thermally treated mortar	4.71	26.39	5.57	0.28	58.93	7.91	1.64	0.91	0	0	0	0.87	0
mortar with oil	4.79	3.70	0.77	0.41	90.80	0	0	0	0.062	0	0.047	0.15	0.023

Similarly, high efficiency in the conversion of *Jatropha curcas* oil with elevated FFA content into biodiesel has been attributed to the catalyst derived from calcined cornstalk powder. This material is notably rich in basic oxides, particularly CaO (13.01%), MgO (3.69%), and K_2_O (38.30%), which provide essential active sites for FFA neutralization through ionic interactions and promote transesterification reactions.^29^

Figure 2 presents a comparative analysis of the interaction between FFA and mortar samples under three conditions: *in natura*, thermally treated at 850 °C and thermally treated at 850 °C oil-saturated. The Ca/Si, Mg/Si, and COH ratios were used as indicators of chemical composition and the availability of reactive sites. The 3D chart highlights variations in the relative intensity of these parameters among the different mortar states.

**Figure 2:**
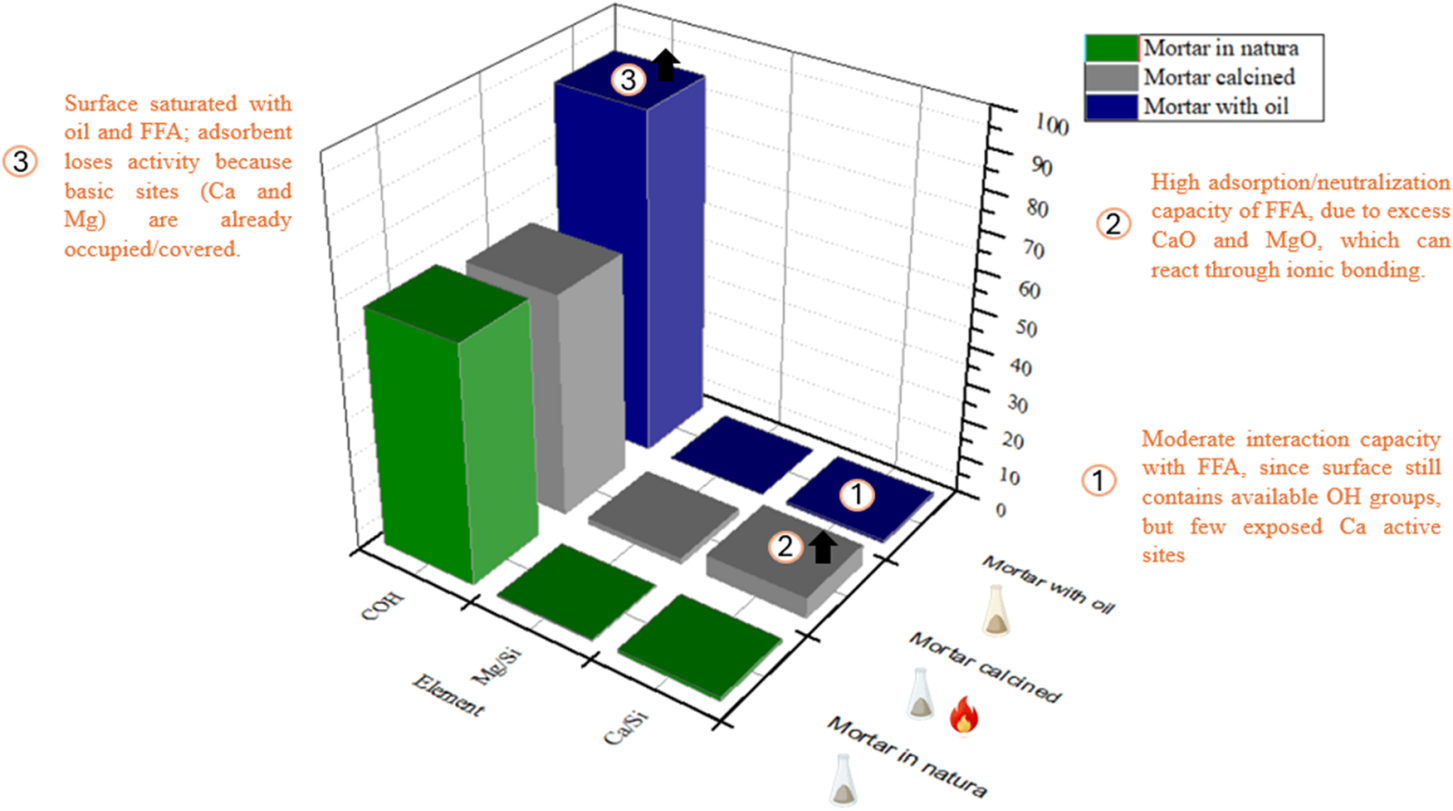
Comparative effect of Ca/Si, Mg/Si, and COH ratios on free fatty acid (FFA) interaction in mortar samples.

The calcined mortar exhibited the highest potential for the FFA adsorption or neutralization. This was attributed to the formation of basic oxides (CaO and MgO) during calcination, which increased the availability of active sites capable of reacting through ionic interactions. In contrast, the natural mortar showed a moderate interaction capacity, mainly associated with the presence of surface hydroxyl groups (COH).

Conversely, the oil-saturated mortar showed a significant reduction in the interaction activity. The surface became coated with oil and FFA, which led to the blockage of Ca- and Mg-active sites and limited further adsorption. These results indicate that the chemical state of the mortar directly controls the availability of reactive sites and their interaction with organic compounds.

Thermogravimetric analysis of the adsorbent was performed to verify the degradation profile of the untreated mortar as a function of temperature variation (Figure 3). The behavior observed in Figure 3 indicates that the first mass loss, corresponding to free water in the temperature range of 25–105 °C, was 1.8%. An additional mass loss of approximately 12% was observed, which demonstrates a slightly higher organic matter content. A study using construction waste, such as white cement rich in calcium,^30^ reported a total mass loss of 10.53%, which is similar to the findings of this present study.

**Figure 3:**
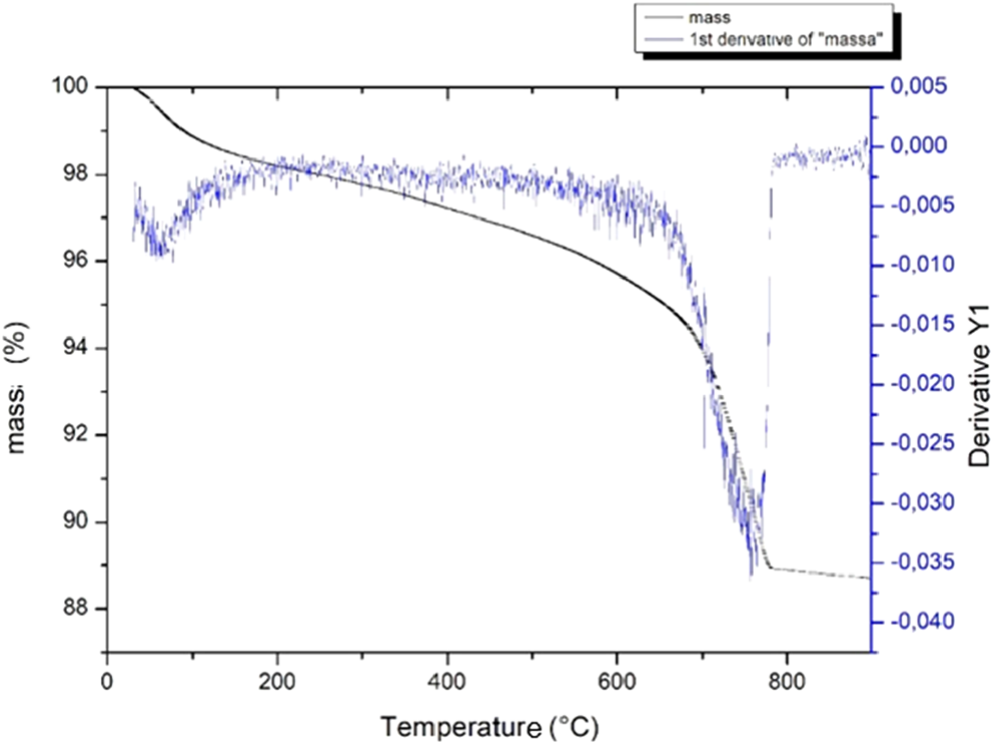
TGA thermograms of untreated mortar residue (in natura).

The second mass loss, recorded in the range of 105–670 °C, was approximately 4.23%, and likely corresponds to the dehydroxylation of portlandite. At approximately 750 °C, the most significant mass loss was attributed to the thermal decomposition of calcium carbonate (CaCO_3_ → CaO + CO_2_), indicating the presence of carbonate phases in the sample.^26,31–33^ This corroborates the fact that, at higher temperatures, the material develops a more porous structure, which facilitates the availability of active sites for adsorption in heat-treated materials.^26^

### 3.2 Morphological Analysis by Scanning Electron Microscopy (SEM)

The analysis demonstrated that heating concrete powder to around 800 °C promoted the release of Ca^2+^ ions, which facilitated the formation of a calcium-rich surface layer and the precipitation of calcium phosphates. In this temperature range, the aluminosilicate phases normally did not react extensively with Ca­(OH)_2_, mainly due to the CO_2_ release resulting from CaCO_3_ decomposition. This CO_2_ evolution contributed to the development of a more porous structure, which remained supported by the original silicoaluminate framework.

The mortar treated at 850 °C maintained its structural composition and primary mineral composition, as indicated by the SEM and EDS results (Figure 4). These findings suggested that the material retained significant potential to act as reactive adsorbent for organic molecules. Elements such as silicon (Si) and aluminum (Al), related to C–S–H and C–A–S–H phases, remained stable after thermal treatment, suggesting a structural rearrangement of the silicoaluminate network rather than complete degradation. Calcium (Ca), initially present as hydroxide or carbonate,^33,34^ was converted into CaO, which improved the release of Ca^2+^ ions and enhanced surface reactivity, a key factor in interactions with acidic substances such as free fatty acids. Furthermore, all of the ternary geopolymer mortar mixes exhibited excellent thermal stability up to 900 °C.^35^

**Figure 4:**
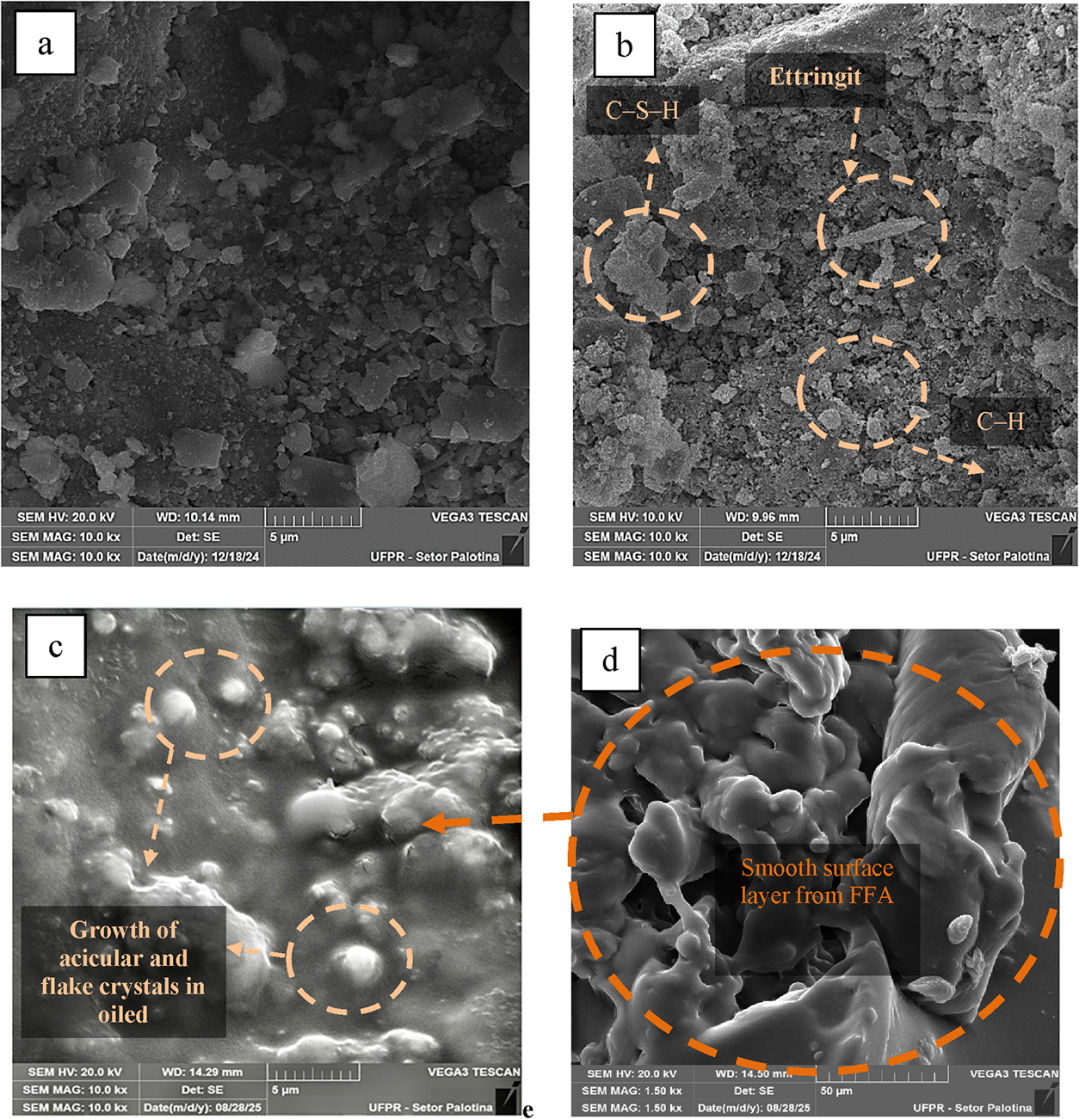
SEM micrographs of the modified mortar surfaces: (a) untreated mortar, in nature (reference sample); (b) mortar thermally treated at 850 °C, showing hydration products with morphologies associated with ettringite-like acicular structures and C–S–H gel regions; (c) thermally treated mortar after FFA adsorption, exhibiting agglomerated acicular and plate-like precipitates distributed over the matrix surface; and (d) higher-magnification image of the adsorbed sample, highlighting the formation of a compact and relatively homogeneous surface layer partially covering the matrix and pore structure.

The SEM micrographs revealed significant microstructural modifications in the mortar surface after thermal treatment and subsequent FFA adsorption. The untreated mortar (Figure 4a) exhibited a heterogeneous and porous cementitious matrix with irregularly distributed hydration products. After thermal treatment at 850 °C (Figure 4b), the matrix presented partially densified regions containing morphologies typically associated with C–S–H gel, hexagonal calcium hydroxide (CH) crystals, and needle-like ettringite structures, which are commonly reported in thermally modified cementitious systems. Following FFA adsorption (Figure 4c), the mortar surface showed the formation of acicular and flake-like precipitates together with agglomerated deposits distributed over the cementitious matrix, indicating the development of secondary surface products and partial pore coverage. At higher magnification (Figure 4d), a compact and relatively continuous superficial layer was observed partially covering pores and pre-existing hydration products, suggesting surface deposition and pore-filling effects after the adsorption process, consistent with densification mechanisms reported for modified cementitious matrices.

Pahlavan and collaborators^36^ observed similar formations, such as the development of acicular and flake-like crystal structures in lime–brick mortars treated with oil-based additives. These formations contributed to enhanced microstructural consistency. Similarly, a study^37^ reported that carboxylic acid derivatives are capable of modifying the morphology of lime–pozzolanic hydrated phases, although this effect was not observed in mortars made with nonhydraulic (air) lime. In this present study, the incorporation of WCOs into mortar mixtures led to notable hydrophobic properties, including a 75–77% decrease in water absorption and a 95% reduction in capillary uptake. Additionally, the oil-treated mortars exhibited significantly improved surface durability when compared to control samples.

### 3.3 Thermal Effects on Functional Groups and Crystalline Phases of Mortar Waste: An FTIR–XRD Comparative Approach

FTIR spectroscopic analysis (Figure 5) allowed the identification of the main differences among three types of mortar: untreated (*in natura*), thermally treated, and thermally treated at 850 °C after FFA adsorption. The interpretation was based on typical wavenumber ranges associated with functional groups found in both inorganic and organic phases.

**Figure 5:**
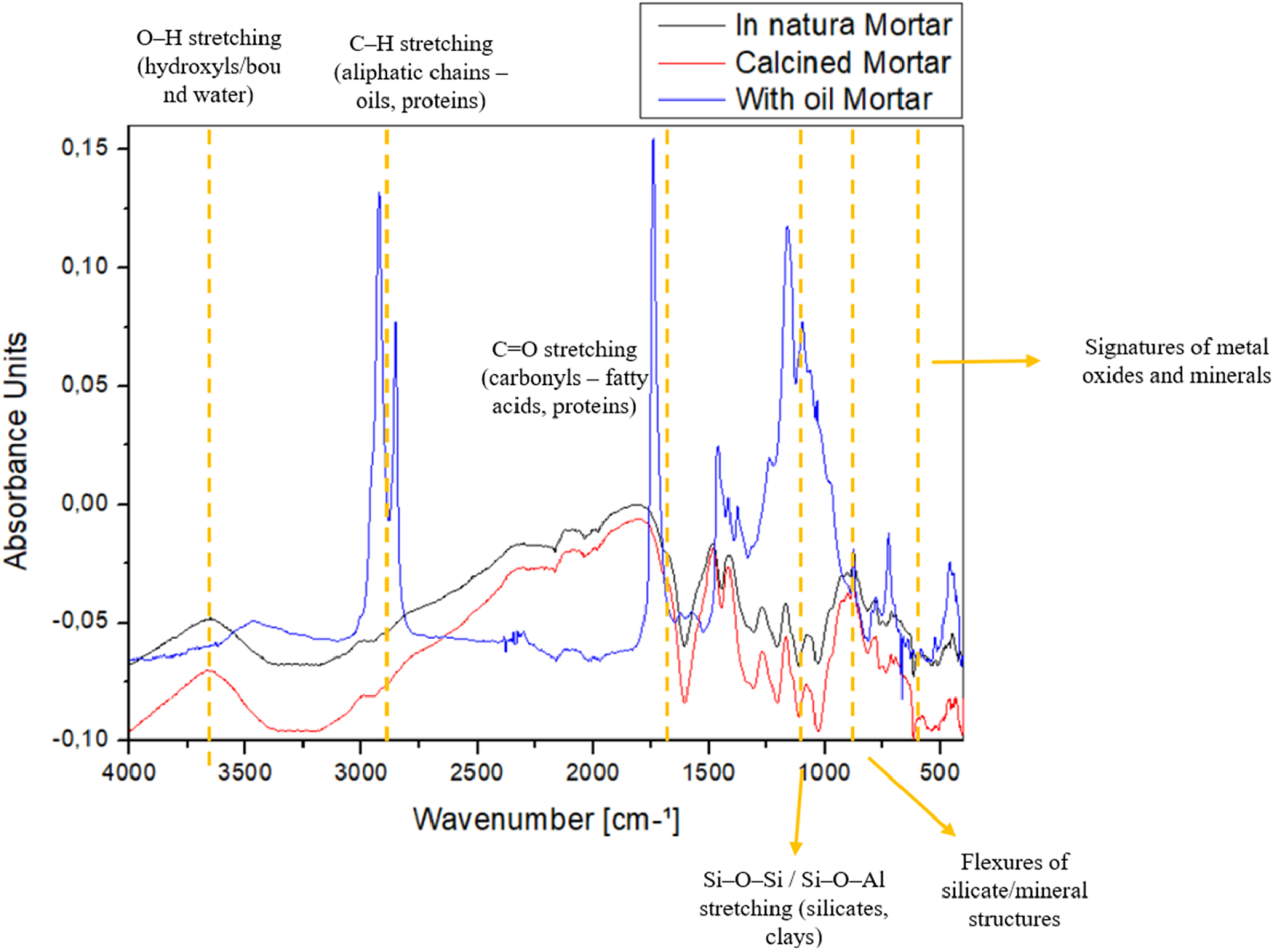
Comparative FTIR spectra of mortars: thermally treated at 850 °C; thermally treated at 850 °C after adsorption of FFA.

The FTIR spectra of the *in natura* and the calcined mortar (treated at 850 °C) showed clear evidence of chemical and structural changes caused by the thermal treatment (Figure 5). The broad O–H band, observed between 3700 and 3000 cm^–1^, typically indicates the presence of hydroxyl groups. This band was strongly reduced after calcination, suggesting the loss of structural water. The carbonate peak, around 1500–1400 cm^–1^, also decreased, indicating the decomposition of CaCO_3_ into CaO and CO_2_. The silicate bands (1100–900 cm^–1^) remained in both samples, although some shifts occurred, indicating that there was some rearrangements in the silicoaluminate structure. Below 800 cm^–1^, the appearance of new bands suggested the formation of Ca–O and potentially Mg–O bonds. These alterations confirmed that calcination not only promoted the removal of water and carbonates but also induced modifications in the mineral structure, increasing porosity and the number of basic sites. These aspects help explain the affinity for FFAs and other similar molecules.

The inorganic matrix of all mortars was characterized by the stretching vibrations of silicates and aluminosilicates (Si–O–Si/Si–O–Al) at approximately 1000 cm^–1^, complemented by mineral bending modes and metal oxide signatures in the 900–400 cm^–1^ region. This interpretation is supported by the literature, which confirms the asymmetric stretching range of Si–O–T (where T = Si or Al) between 830 and 1200 cm^–1^ in cementitious binders,^38^ as well as by studies that have employed FTIR for the quantitative mineralogical analysis of historical mortars.^39^

The primary distinction was found in the oil-treated sample, which displayed a strong signature of organic additives. Intense absorption peaks in the C–H stretching region (2900 cm^–1^) and the C=O stretching region (1700 cm^–1^) indicated the incorporation of aliphatic chains, fatty acids, or proteinaceous compounds. This finding is widely corroborated in the literature, where the C–H and C=O bands serve as primary markers for identifying organic additives (oils, fats, or gums) in mortars, historically used to enhance cohesion, durability, or water resistance.^40,41^

The Fourier transform infrared (FTIR) spectroscopic analysis of the mortar samples, encompassing the material in natura, thermally treated at 850 °C, and post-FFA (free fatty acid) adsorption, identifies the key mineralogical phases through their diagnostic vibrational bands. The low-frequency region is characterized by Al–O and Al–OH stretching and bending modes appearing at 847–848 cm^–1^,^42^ alongside Fe–O vibrational modes localized between 500 and 700 cm^–1^.^43^

The structural silicate network SiO_2_ is evidenced by absorption bands in the 850–935 cm^–1^ range^43^ and further confirmed by vibrations in the 1011–1080 cm^–1^ region.^46^ Regarding the presence of oxyanions, sulfate groups (SO_4_^2–^) were identified within the 1100–1200 cm^–1^ interval,^44^ while the carbonation of the matrix is manifested by the characteristic carbonate CO_3_^2–^ bands occurring between 1400–1500 cm^–1^ and 1795–1996 cm^–1^.^45^

The calcium-bearing phases are represented by two distinct signatures: the stretching vibrations of Ca­(OH)_2_ (portlandite) at 3641–3644 cm^–1^ 44 and the presence of CaO (calcium oxide) at 540–550 cm^–1^,^42^ the latter of which is particularly significant for characterizing the effects of thermal treatment on the mortar samples.

Figure 6 presents the XRD spectra for *in natura*, thermally treated mortar (850 °C), and thermally mortar treated at 850 °C after FFA adsorption. The *in natura* mortar exhibited the highest crystallinity, as indicated by sharp and intense peaks. The thermally treated mortar also displayed defined peaks but with reduced intensity. In contrast, the oil-treated mortar exhibited significantly lower peak intensity, suggesting a decrease in crystallinity likely caused by the presence of organic compounds.

**Figure 6:**
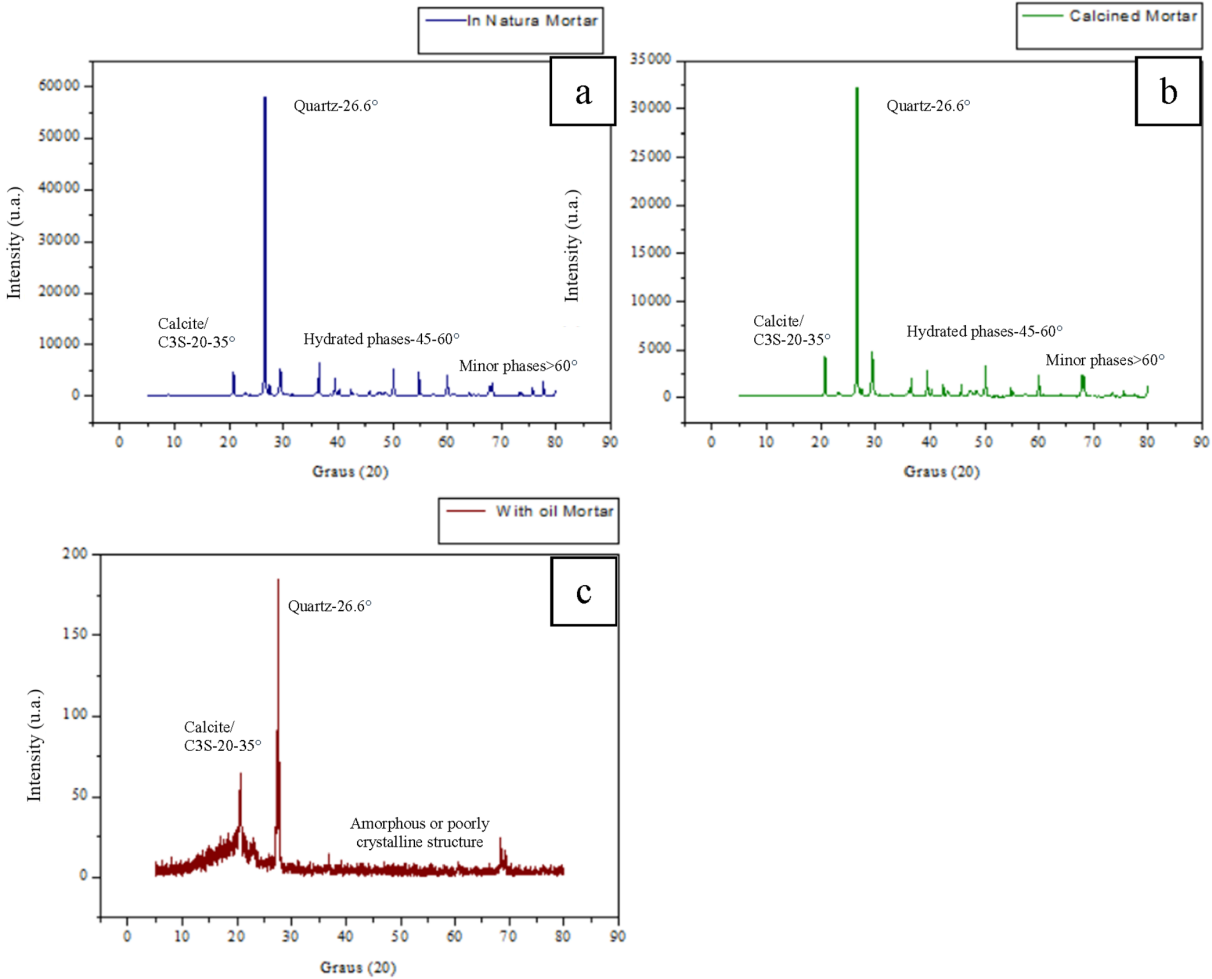
X-ray diffraction (XRD) patterns of mortar samples: (a) untreated mortar (in natura), (b) mortar calcined at 850 °C, and (c) calcined mortar after oil adsorption.

The most intense peaks were observed at a 2θ angle between 25° and 30°, indicating the presence of a calcium silicate phases, particularly wollastonite (CaSiO_3_). This suggests that both adsorbents share a similar mineralogical composition, predominantly composed of calcium silicates (CaSiO).^47^

In the 2θ range between 30° and 90°, the thermally treated mortar exhibited sharp, well-defined peaks, indicating highly crystalline phases, likely calcium silicates and aluminates formed or stabilized by heat treatment. This confirms that calcination promoted the crystallization or recrystallization of mineral phases. On the other hand, the oil-treated mortar exhibited a broad, low-intensity pattern with few peaks, reflecting an amorphous or poorly crystalline structure. The presence of organic compounds, such as WCA, likely disrupted crystal formation, inhibited phase growth or encapsulated mineral precursors.

### 3.4 Modeling and Optimization of Free Fatty Acid Reduction

As shown in Figure 7, lower temperatures combined with moderate agitation levels led to better acid reduction. Interestingly, higher temperatures with higher agitation also resulted in a strong reduction in the acidity of the residual frying oil.

**Figure 7:**
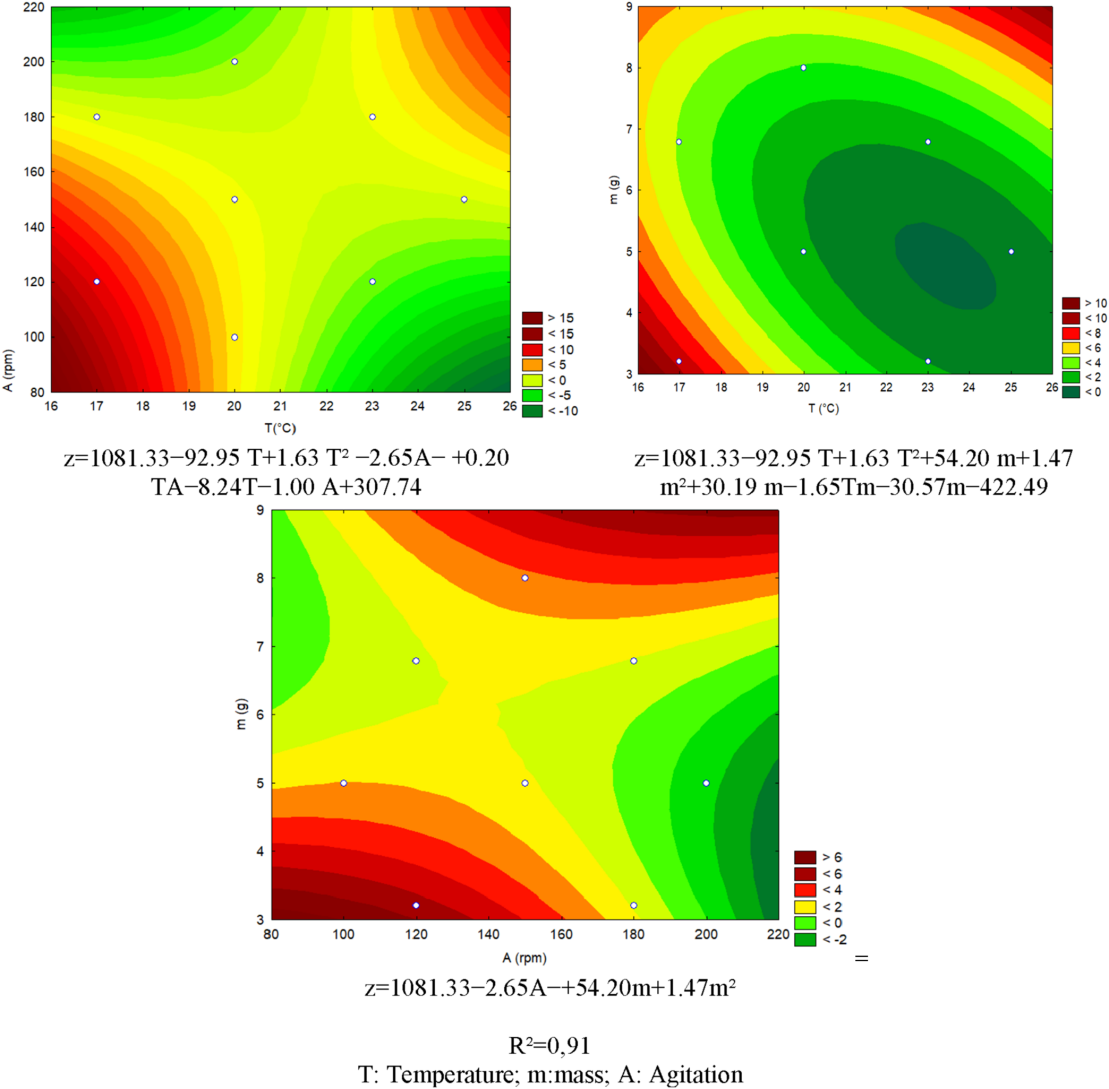
Free fatty acid reduction response surface as a function of temperature, agitation, and mass using thermally treated mortar at 850 °C.

According to the ANOVA results (Table 5), temperature, agitation, and mass had significant linear and/or quadratic effects on acidity reduction (*p* < 0.05), except for the quadratic effect of agitation. All of the two-factor interactions were also significant. The lack of fit was not significant (*p* = 0.0748), indicating that the model adequately described the experimental data. The root-mean-square error (RMSE) was 4.97.

**Table 5:** Analysis of Variance (ANOVA) for the Response Variable: Acidity Reduction

source of variation	SS	DF	MS	*F*-value	*F*-critical (α = 0.05)	*p*-value
temperature (linear)	815.219	1	815.219	330.216	7.71	0.004546
temperature (quadratic)	1.353,093	1	1.353,093	548.088	7.71	0.001776
agitation (linear)	765.662	1	765.662	310.144	7.71	0.005093
agitation (quadratic)	4.547	1	4.547	0.1842	7.71	0.689894
mass (linear)	693.005	1	693.005	280.711	7.71	0.006095
mass (quadratic)	264.857	1	264.857	107.283	7.71	0.030632
1L × 2L interaction	1.649,460	1	1.649,460	668.136	7.71	0.001220
1L × 3L interaction	454.199	1	454.199	183.975	7.71	0.012753
2L × 3L interaction	602.541	1	602.541	244.067	7.71	0.007815
lack of fit	377.418	3	125.806	50.959	6.59 (DF1 = 3, DF2 = 4)	0.074829
pure error	98.750	4	24.688	—	—	—
**total**	**5.590.235**	16	—	—	—	—
**RMSE**						**4.97**

These results highlight a synergistic effect between temperature and agitation during the neutralization process, where higher temperatures improved molecular dispersion, while agitation facilitated even particle distribution and reduced resistance in the system.^48^

The data in Figure 7 showed how temperature and the amount of adsorbent interact. Notably, even at lower adsorbent amounts, increasing the temperature still leads to acid reduction. Conversely, higher adsorbent dosages at lower temperatures also led to effective performance. In this study, a 78% reduction in the acid value was achieved under the conditions of 20.29 °C, 153.53 rpm, and 5 g of adsorbent using calcined mortar. Even with a small amount and moderate temperature, a substantial decrease in acidity was observed. These findings are similar to those in the literature,^49^ where the authors reported an acid index of 51.47% using 2 g of adsorbent, 125 rpm, and 18 °C. According to them, the high acidity is attributed to the hydrolysis of fats caused by water released from frozen foods during frying. Although their process was effective, it required a higher amount of adsorbent. In comparison, the present results demonstrate that, under optimized conditions, effective acidity reduction can be achieved with lower material consumption, improving process efficiency and reducing operational costs.

The quadratic effect of temperature was significant, suggesting that the relation between temperature and acid reduction is nonlinear. There is probably a certain range where the effect is better. While the linear terms of agitation and adsorbent mass contributed positively to the process, the quadratic term for the agitation was not significant. This indicates that variations in agitation did not significantly affect the response within the experimental ranges evaluated (Figure 8).

**Figure 8:**
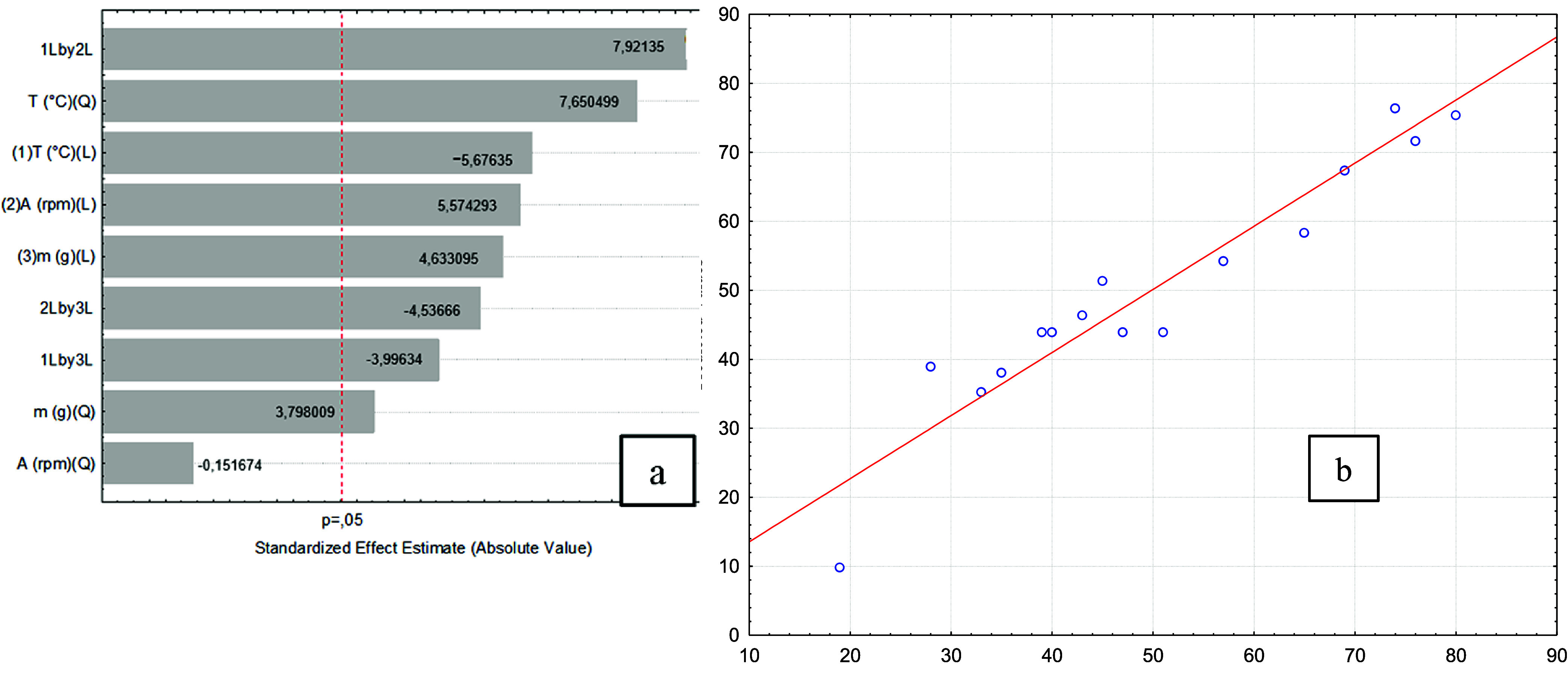
Pareto chart of standardized effects for the reduction of free fatty acid content (a). Observed vs predicted values for acidity reduction (b).

As shown in Figure 9, FFA reduction increased as a function of contact time for the mortar thermally treated at 850 °C. The removal efficiency rose gradually between 5 and 120 min, reaching approximately 78% at the longest evaluated time, which indicates progressive occupation of active sites until near-equilibrium conditions were established. No abrupt decrease was observed. Instead, the data suggest a kinetic trend of continuous uptake followed by stabilization. In parallel, the pH remained in the alkaline range (8–11.5), confirming the chemical stability of the material during treatment and supporting its role in promoting acid reduction.

**Figure 9:**
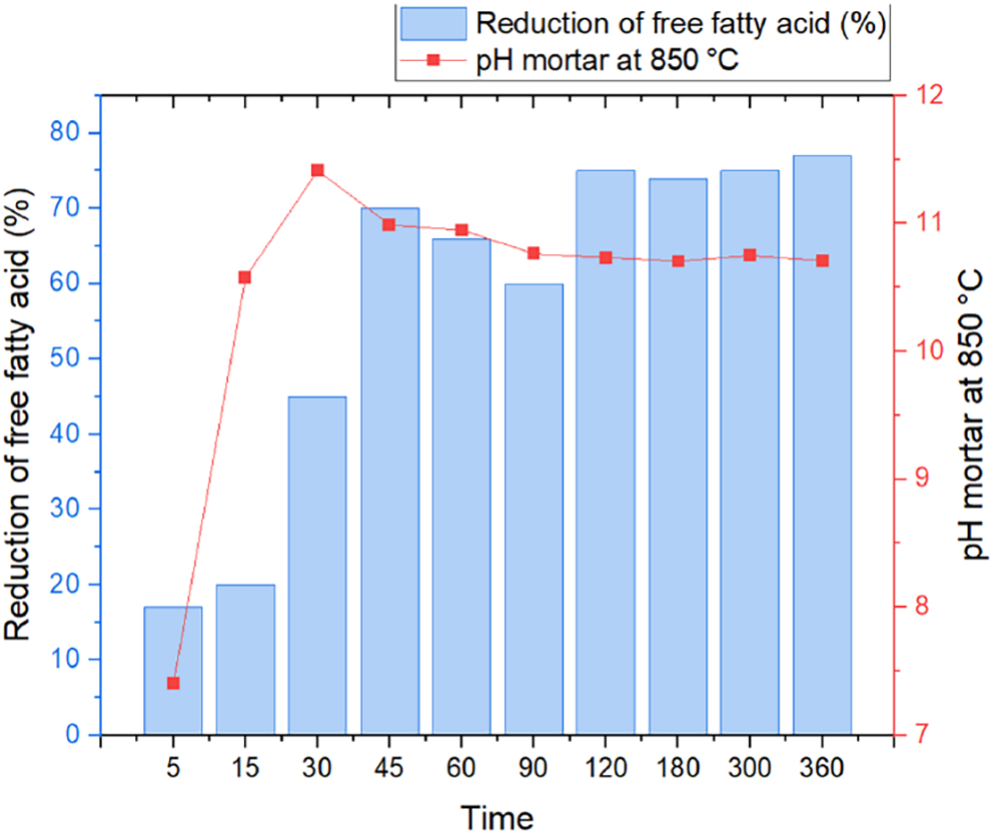
Effect of contact time on free fatty acid reduction and pH variation of thermally treated mortar at 850 °C.

### 3.5 Mechanistic Insights from Adsorption Kinetics

The experimental data for the FFA adsorption capacity (qt) onto the calcined residue surface are presented in Figure 10. The results were fitted by using four classic kinetic models: Elovich, pseudo-first-order (PFO), pseudo-second-order (PSO), and intraparticle diffusion (IPD). The PSO model provided the best correlation with the experimental data, indicating that the rate-limiting step is predominantly governed by chemisorption. This suggests that the adsorption process involves electron exchange or sharing between the adsorbate (FFA) and the active sites on the heterogeneous adsorbent surface, consistent with the high surface basicity observed in calcined cementitious residues.

**Figure 10:**
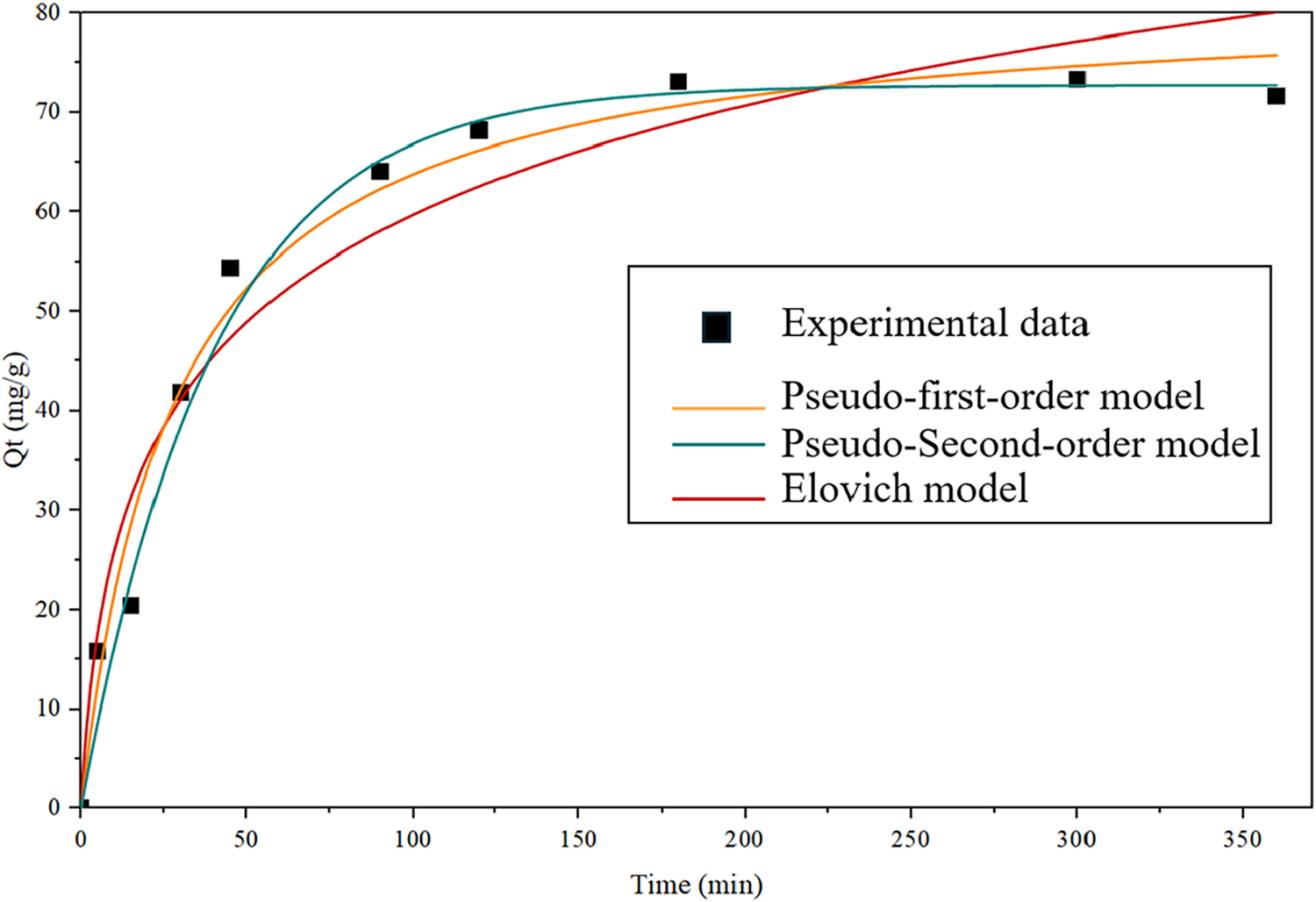
Kinetic modeling of free fatty acid (FFA) adsorption of thermally treated mortar at 850 °C: experimental data and model fits (Elovich, Pseudo-First Order, Pseudo-Second Order, and Intraparticle Diffusion).

The Elovich model demonstrated satisfactory alignment during the initial adsorption stages, suggesting that the system exhibits energetically heterogeneous adsorption sites and a rapidly declining adsorption rate due to progressive site deactivation. This behavior is characteristic of systems in which physisorption transitions to chemisorption as the process evolves. In contrast, the PFO model underestimated the equilibrium adsorption capacity, particularly at longer contact times, indicating that the assumption of a monolayer physisorption mechanism with uniform active sites is insufficient for describing the experimental system (Table 6).

**Table 6:** Kinetic Modeling Parameters for FFA Adsorption of Thermally Treated Mortar at 850 °C: Comparison of PSO, PPO, and Elovich Models

model	parameter *A*/*q*_e_	parameter *B*/*k*_2_/*K*_diff_	*R* ^2^	adjusted *R* ^2^
PSO	81.66796 ± 3.08514	4.37165 ± 8.16686 × 10^–5^	0.98127	0.97893
Elovich	6.3825 ± 2.6614	0.06201–6 ± 0.009	0.94908	0.94271
PPO	72.75552 ± 0.0982	0.02511 ± 1.84496 × 10^–4^	0.97318	0.97315

The obtained results reinforce the technical feasibility of using thermally activated mortar waste as an effective adsorbent for the pretreatment of WCOs with a high FFA content. This is an essential step to ensure the economic viability of the biodiesel production. Kinetic analysis revealed that the PSO model provided the best fit (*R* ^2^ = 0.96786), indicating that the adsorption process was primarily governed by chemisorption. Similar behavior was reported in the literature where a 72% FFA reduction in WCO using quicklime,^50^ and over 70% FFA removal was obtained using activated charcoal derived from avocado seeds.^51^ Additionally, alternative materials such as coal ash^52^ and biomass-derived shells^53^ have also demonstrated high performance in FFA adsorption. These findings suggest that both mineral- and biomass-derived adsorbents, when thermally activated, provide high efficiency in acidity reduction with low cost and environmental impact, presenting a promising alternative to conventional acid esterification pretreatment methods.

## Conclusion

This study demonstrated that thermally treated mortar residues derived from construction and demolition waste can be effectively employed as low-cost adsorbents for the reduction of acidity in WCOs with high FFA content. Thermally treated at 850 °C induced significant physicochemical modifications in the material, promoting the formation of basic oxides, such as CaO and MgO, which serve as active adsorption sites. Consequently, the treated mortar exhibited high reactivity, achieving an acidity index reduction of up to 78% under optimized conditions.

Kinetic analysis indicated that the PSO model provided the best description of the adsorption behavior, suggesting that the process is predominantly controlled by chemisorption mechanisms associated with the interaction between basic sites of the adsorbent and acidic compounds present in the oil. These findings confirm the effectiveness of thermally activated mortar residues as functional materials for the pretreatment of low-quality lipid feedstocks.

From an application perspective, the reduction of FFA content is essential for biodiesel production, since values above 4% favor saponification during transesterification, which decreases biodiesel yield and increases downstream purification costs. Therefore, the proposed approach represents a promising strategy for simultaneously valorizing construction waste and improving the processing of WCOs, contributing to circular economy practices in both the construction and biofuel sectors. Future investigations should focus on adsorption equilibrium behavior, including isotherm modeling, as well as regeneration and reuse of the adsorbent to further assess its industrial feasibility.
